# Do four or more antenatal care visits increase skilled birth attendant use and institutional delivery in Bangladesh? A propensity-score matched analysis

**DOI:** 10.1186/s12889-019-6945-4

**Published:** 2019-05-16

**Authors:** Bridget L. Ryan, Rohin J. Krishnan, Amanda Terry, Amardeep Thind

**Affiliations:** 1 Department of Epidemiology and Biostatistics, Western University, 1151 Richmond Street, London, ON Canada; 2 Department of Family Medicine, Western University, London, ON Canada; 3 Schulich Interfaculty Program in Public Health, Western University, London, ON Canada

**Keywords:** Antenatal care, Skilled birth attendant, Institutional delivery, Propensity score matching, And Bangladesh

## Abstract

**Background:**

With Bangladesh’s adoption of the third Sustainable Development Goal to reduce maternal mortality, the impetus for Bangladesh to continue to improve uptake of maternal healthcare is strong.

**Methods:**

Using a propensity-score matched analysis, the present study utilized data from the 2014 Bangladesh Demographic Health survey to examine the impact of four or more antenatal care visits on skilled birth attendant use and institutional delivery.

**Results:**

The results revealed a significant and positive impact of four or more antenatal care visits on skilled birth attendant use and institutional delivery after matching treated and untreated mothers on included socio-demographic characteristics.

**Conclusions:**

Implementation of policies to provide at least four antenatal care visits may serve as an effective strategy to increase SBA use and institutional delivery in Bangladesh, which could contribute to the reduction of maternal mortality.

**Electronic supplementary material:**

The online version of this article (10.1186/s12889-019-6945-4) contains supplementary material, which is available to authorized users.

## Background

The global community has adopted Sustainable Development Goals of which goal three (SDG-3) aims to reduce the worldwide maternal mortality ratio (MMR) to 70 maternal deaths per 100,000 live births by 2030 [1]. From 1990 to 2015, Bangladesh’s MMR declined substantially from 569 to 176 deaths per 100,000 live births - a 69% reduction in the MMR [1, 2].

As advocated by the World Health Organization (WHO), increased uptake of skilled birth attendants (SBAs) and deliveries within a medical institution is of paramount importance in reducing maternal mortality [3]. Extensive research has sought to understand the various socio-demographic correlates associated with SBA use and institutional delivery in Bangladesh [4]. In particular, recent evidence suggests an association between the use of antenatal care (ANC) and both SBA use and institutional delivery [5–7]. However, ANC in and of itself may not directly result in SBA use and institutional delivery uptake; rather it may be that mothers who utilize ANC may differ across known factors to influence SBA use and institutional delivery; that is, they may be more educated, wealthy, or have more media exposure [8].

Efforts to control for such confounding associations between ANC use, SBA use, and institutional delivery in statistical analyses have traditionally been accomplished via regression analyses. However, bias may inevitably still persist. For example, the positive effects of antenatal care on SBA use and institutional delivery may be biased because the distribution of factors influencing ANC use among mothers who use ANC compared to mothers who do not may still differ, even when such characteristics are controlled for within regression models [8–11]. Furthermore, women who use ANC may inherently be more risk averse as compared to women who do not use antenatal care [8]. Such unobservable characteristics can also introduce bias [8].

Propensity score matching is a methodological technique that endeavors to remove bias by matching treated and untreated individuals with similar conditional probabilities to receive the treatment [10, 12]. In this study, we matched mothers who used ANC to mothers who did not use ANC with similar propensity score values for ANC use [8]. It can then be reasoned that any difference in SBA use or institutional delivery among mothers in the matched sample can be attributed to the utilization of ANC alone. The use of propensity scores has garnered support and its proponents regard it as a preferred method compared to traditional regression adjustments, such as logistic regression [12, 13]; however we identified no studies in Bangladesh that used propensity scores to study the impact of ANC on maternal healthcare utilization. The present study addresses the methodical limitations of previous studies by examining the effect of ANC on SBA use and institutional delivery among mothers in Bangladesh using propensity score matching (PSM) analysis.

## Methods

This study was reported in agreement with The Strengthening and Reporting of Observational Studies in Epidemiology (SROBE Statement) for propensity score analyses [14, 15].

### Dataset and key variables

The 2014 Bangladesh Demographic Health Survey was used for this analysis. Detailed information regarding sampling design and response rates can be found elsewhere [16]. Data from the BDHS were obtained from in-person interviews. The sample was limited to the most recent pregnancy of married mothers who had given birth within three years preceding the time of the interview (*N* = 4441). Two maternal healthcare utilization outcomes were chosen: SBA use and institutional delivery. SBA use was a dichotomous variable where respondents were coded as having delivered by an SBA if they received delivery care by a qualified doctor, nurse, midwife, paramedic, family welfare visitor or community skilled birth attendant. Mothers were considered as having an institutional delivery if they delivered within a public, private, or non-governmental organization (NGO) facility. The treatment variable (i.e. exposure of interest) was dichotomous with ‘Appropriate ANC’, defined as the receipt of at least four ANC visits; this definition was guided by WHO recommendations at the time of the survey [17]. Women who received no ANC visits or 1 to 3 visits were considered as the non-exposed group, not having received Appropriate ANC. Only six missing values were recorded for appropriate ANC. Regarding the outcome variables, institutional delivery had two missing values.

#### Propensity Scores and Average Treatment Effects

A propensity score is defined as the, “conditional probability of assignment to a particular treatment given a vector of observed covariates” (Rosenbaum & Rubin, 1983, pg.41) and is expressed as follows:

$$ \mathrm{p}\left(\mathrm{X}\right)=\mathrm{pr}\left(\mathrm{D}=1|\mathrm{X}\right) $$

where is p(X) is the conditional probability of receiving a minimum of four ANC visits, D = (0,1) is exposure to appropriate ANC, and X is the vector of chosen socio-demographic characteristics correlated with appropriate ANC and SBA use and institutional delivery [9, 18].

The estimation of average treatment effects on the treated (ATT) follows a counterfactual framework and can be expressed as follows:

$$ \mathrm{ATT}=\mathrm{E}\left({\mathrm{Y}}_{1\mathrm{i}}|{\mathrm{D}}_{\mathrm{i}}=1\right)-\mathrm{E}\left({\mathrm{Y}}_{0\mathrm{i}}|{\mathrm{D}}_{\mathrm{i}}=1\right) $$

where E(Y_1i_| D_i_ = 1) is the expected outcome of SBA use and institutional delivery if all treated mothers received appropriate ANC and E(Y_0i_| D_i_ = 1) is the expected outcome of SBA use and institutional delivery among treated mothers had none of the mothers received appropriate ANC (unobserved) [8, 10, 19].

Thus, the ATT can be interpreted as the average difference in SBA use and institutional delivery that would be found if all treated mothers received appropriate ANC compared to the same individuals had they not used appropriate ANC [10].

Given that it is not possible to observe individual treatment effects among treated mothers if they had used and not used appropriate ANC simultaneously [10], we constructed the counterfactual using propensity scores by ‘matching’ mothers who received appropriate ANC to mothers who did not receive appropriate ANC on a set of observable characteristics [8]. That is, mothers who did not receive appropriate ANC served as a counterfactual case to treated mothers had they not used appropriate ANC [8].

### Statistical analysis

Descriptive analysis was first undertaken to examine the prevalence of SBA use and institutional delivery according to the number of ANC visits received. Next, Stata’s pscore command was used to generate the propensity score using a logit model fitted using covariates associated with the receipt of at least four ANC visits (referred to as Appropriate ANC) and both outcomes (Table 1) [20]. Selection of relevant variables was guided by Andersen’s Behavioral Model of Health Services Utilization [21] and prior literature. Variables were chosen only if they were conceptualized to occur prior to treatment assignment (Table 1).

**Table 1:** Variables Definitions

Age (years)	Continuous
Birth Order	Continuous
Religion	0. Non-Muslims 1. Muslims
Maternal/Husband’s Formal Education	0. No formal education 1. Primary Education 2. Secondary or Higher Education
Maternal Literacy	0. Could not read sentence or could only read part of a sentence 1. Can read whole sentence
Wanted Pregnancy	0. Wanted their pregnancy no more 1. Wanted their pregnancy then or later
Media Exposure	0. Does not watch television, read newspaper, or listen to radio at least once a week 1. Exposed to one media (tv, newspaper, or radio) outlet at least once a week 2. Exposed to two or three media (tv, newspaper, or radio) outlets at least once a week
Autonomy	0. No ‘say’ in making large household purchases, visiting family, and decisions regarding her own healthcare 1. Has say in one indicator 2. Has say in two indicators 3. Has say in three indicators
Location	0. Rural 1. Urban
Household Wealth Index	1. Poor & Poorest 2. Intermediate 3. Rich & Richest
Lost Pregnancy prior to most recent pregnancy	0. No 1. Yes
Skilled Antenatal Care Provider	0. No 1. Yes

Note: All variables, apart from age and birth order, were left categorical when generating the propensity score. Missing values were negligible. Husband’s education had two missing values and maternal literacy had only one missing value

The common support option was employed to limit testing of the balancing property to only treated mothers whose propensity score for SBA use and institutional delivery lay within the range of propensity scores (i.e. above minimum and below max) for controls. Using Stata’s pstest command to measure covariate balance, we tested the following matching methods: nearest neighbor matching with and without replacement, and radius matching with calipers from 0.01 to 0.05. Stata’s psmatch2 command was used to generate the ATT for the matching method that produced the highest quality of matches. The common support option was also employed to produce higher quality matches.

An important assumption of PSM is uncounfoundedness which requires that there is no unmeasured selection bias influencing treatment selection. Since it is not possible to directly test this assumption, an alternative approach determines the degree to which significant results rely on this assumption being true. This was accomplished through a sensitivity analysis where we varied the odds (as measured by gamma) for differential assignment to the treated group due to an observed variable, and then inspected the accompanying significance bounds to determine the degree of selection bias needed to change a significant treatment effect to non-significance [22]. For this study, the magnitude of gamma imposed on the results ranged from 1 to 2 via 0.5 increments.

## Results

**Fig. 1:**
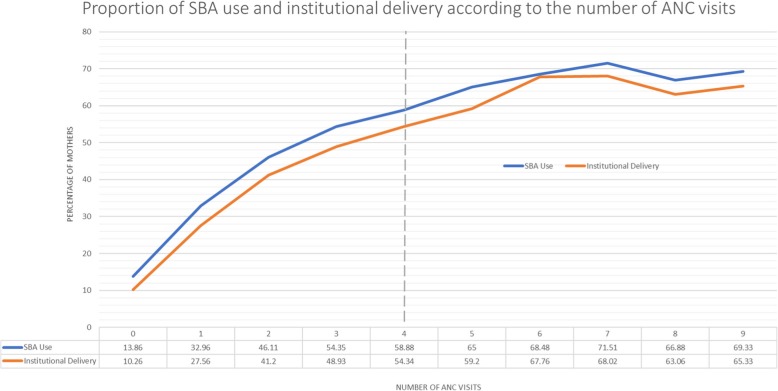
Percentage of mothers using a skilled birth attendant and having an institutional delivery according to the number of ANC visits they received

Descriptive analysis revealed that 32% of women received four or more ANC visits; 47% received 1 to 3 ANC visits; and 21% of women received no ANC. The proportion of women reporting SBA use and institutional delivery increased as the number of ANC visits increased. (Fig. 1; Additional file 3 provides the figure with the data table). The percentage increase in SBA use and institutional delivery tended to be highest on average among mothers with zero to four visits.

Radius matching, with caliper of 0.01 produced the highest quality of matches and was the chosen matching method for this analysis. This can be seen through the noticeable reduction in bias between individual variables (Additional files 1 and 2) and the pseudo R^2^ statistic between matched and unmatched mothers, which indicates that the distribution of covariates was similar between treated and untreated mothers in the matched sample (Table 2) [8]. These findings were confirmed through inspection of the *p*-value for the likelihood ratio test for joint significance which was not statistically significant (SBA use, *P* > X^2^ = 0.999; institutional delivery, *P* > X^2^ = 0.999) after matching (Table 2).

**Table 2:** Quality of matching and average treatment effects on the treated (ATT) of appropriate ANC on SBA use and institutional delivery

	Model Diagnostics							
	Pseudo R^2^	LR Chi2	P > Chi2	Mean Bias	Median Bias	ATT	95% CI	*P*-value
Unmatched	0.132	732.47	0.000	29.5	31.6	–	–	–
SBA Use (*N* = 4431)								
Radius, Caliper (0.01)	0.001	5.57	0.999	1.8	1.7	0.12	0.09–0.16	*P* < 0.001
Institutional delivery (*N* = 4429)								
Radius, Caliper (0.01)	0.001	5.48	0.999	1.8	1.7	0.14	0.10–0.17	*P* < 0.001

Note: Standard Errors were bootstrapped with 100 repetitions

After matching, the probability of SBA use and institutional delivery was 12 and 14% higher respectively among treated mothers who received appropriate ANC compared to the same individuals had they not received appropriate ANC (Table 2). The region of common support was robust; only two treated individuals were outside the region of common support. These cases were dropped from the analysis when calculating the ATT.

The results of the sensitivity analysis demonstrated that, with all specified values of gamma, the average treatment effect of appropriate ANC on SBA use and institutional delivery remained statistically significant (p_mh = 0.000). This indicates that the positive treatment effects observed for both SBA use and institutional delivery are insensitive to hidden bias within the range determined by gamma [8, 22].

## Discussion

A review of the literature found few studies that quantified the association between ANC and SBA use or institutional delivery [5–7]. The majority of studies were largely observational and used traditional regression approaches to examine correlates of maternal healthcare utilization [5–7]. To our knowledge, this is the first study in Bangladesh to use propensity score matching to examine the impact of ANC on maternal healthcare utilization.

The present study revealed a significant and positive impact of appropriate ANC on SBA use and on institutional delivery after matching treated and untreated mothers on all included observable characteristics. It should be noted that some variables which would have been appropriate to include in the propensity matching such as timing of ANC visits, location of ANC, quality of ANC, and the particular order of ANC visits were not available in the 2014 BDHS dataset. The results align with previous literature, which highlighted a positive impact of appropriate ANC on SBA use and institutional delivery in Bangladesh [5–7]. Our results agreed with another study linking ANC with institutional delivery in India that used propensity score matching [9]. It is likely that ANC serves as a critical juncture to educate and refer mothers to health facilities [23, 24]. Overall, the findings from this study support the assertion that the provision of ANC is a tangible means to elicit uptake of SBA use and institutional delivery in Bangladesh. However, the results of this study do not conclusively establish causal relationships between appropriate ANC and SBA and institutional delivery given the cross-sectional nature of the BDHS dataset. As well, while propensity matching removes bias on observable characteristics, hidden bias may still persist due to unaccounted confounders which may overestimate the beneficial effects of ANC on SBA use and institutional delivery, albeit the sensitivity analyses conducted herein suggests that the positive effects of ANC are still largely robust.

Data from the BDHS suggests that 32% of women received four or more ANC visits; there are still 21% of women who received no ANC, 47% received 1 to 3 ANC visits. Given the relationship of ANC to SBA and delivery (both of which are associated with improved maternal and child outcomes), there is an urgent need for Bangladesh to increase the number of women who receive appropriate ANC.

Bangladesh has implemented a number of successful strategies nationally and regionally that could be scaled to reach more women [7, 25–27]. For example, to reduce financial barriers, Bangladesh has implemented maternal health voucher scheme [25, 27]. The voucher scheme targets socioeconomically disadvantaged mothers and provides them with three ANC visits, a SBA for home- or facility-based delivery, one post-natal care visit, and cash remunerations for travel expenses [25]. Past evaluations found the voucher scheme to reduce out-of-pocket expenditures, decrease the equity gap and increase ANC uptake, SBA use, and institutional delivery within select voucher areas [28, 29].

Additionally, in 2007, a regional Maternal Neonatal and Child Health program in Bangladesh was implemented as an extension to an ongoing initiative in rural Matlab [7, 26]. The program enhanced networks between community and facility services. Through this program, existing ANC delivery was also strengthened to include additional evidence-based interventions. The program appears promising as an effective intervention in promoting ANC uptake (see Pervin et al. 2012, Fig. 1), increasing institutional delivery, and reducing perinatal mortality [7, 26].

From a ‘grass roots’ perspective, Bangladesh has also implemented a community health worker program that trains local women as community health workers to educate mothers and deliver important maternity care services including ANC [30]. Importantly, workers help to address the various socio-cultural barriers other community members may face when accessing health services [30]. Given the usefulness of Bangladesh’s community health workforce, the provision of ANC by community health workers may serve as a pragmatic means to bridge cultural differences and further elicit uptake of skilled birth assistance and institutional delivery within the country. Overall, these programs have shown success regionally but it is possible that if they were to be scaled nationally, they may positively impact mothers who are not receiving appropriate ANC.; however, for these programs to be sustainable, they must also be supported with ongoing efforts to strengthen the availability and quality of services as well as reductions in out-of-pocket costs.

The present study provides evidence that four or more visits is sufficient to make a positive impact on two very important pregnancy health care outcomes, SBA and institutional delivery. In September 2015, the WHO advocated for the receipt of eight or more ANC visits, a substantial increase from the previous guideline of a minimum of four visits [31]. Data from the BDHS suggest that the incremental gain in SBA and institutional delivery is highest within the first four ANC visits and then declines (Fig. 1). Given that that 68% of women in Bangladesh receive either zero or fewer than four ANC visits, efforts and resources might be better employed increasing the number of women who receive four visits rather than attempting to provide all women eight or more visits. Future cost-effective analyses are needed to determine the number of ANC visits that will optimize maternal outcomes.

## Conclusion

Overall, the results of this study illustrate a significant and positive association between the receipt of at least four ANC visits on SBA use and institutional delivery among mothers in Bangladesh. Implementation of policies geared towards provision of at least four ANC appointments can serve as an effective and tangible intervention to link mothers to appropriate skilled birth attendants and health facilities for safe motherhood which in turn can assist Bangladesh in meeting the SDG-3 target.

## Additional files

Additional file 1: **Table S1.** Covariate Balance check for SBA use (DOCX 23 kb)

Additional file 2: **Table S2.** Covariate Balance check for Institutional Delivery (DOCX 23 kb)

Additional file 3: **Figure S1.** Fig. 1 with data table (XLSX 15 kb)

## Abbreviations

ANC: Antenatal Care
BDHS: Bangladesh Demographic Health Survey
MMR: Maternal Mortality Ratio
NGO: Non-Governmental Organization
PSM: Propensity Score Matching
SBA: Skilled Birth Attendant
SDG: Sustainable Development Goal
WHO: World Health Organization
