# Supplementary material for: Do four or more antenatal care visits increase skilled birth attendant use and institutional delivery in Bangladesh? A propensity-score matched analysis
Source: BMC Public Health. 2019 May 16;19:583. doi: 10.1186/s12889-019-6945-4 (PMC6521440; doi:10.1186/s12889-019-6945-4)
Supplement: Supplementary file 1 — Table S1. Covariate Balance check for SBA use (DOCX 23 kb) [file 12889_2019_6945_MOESM1_ESM.docx]

**Table S1: Covariate Balance Check for Skilled Birth Attendant use in Bangladesh**

| Variable |  | Mean | | % Bias | % Reduction \|Bias\| | T -Test | |
| --- | --- | --- | --- | --- | --- | --- | --- |
|  |  | Treated | Control |  |  | t | p > \|t\| |
| **Age** | Unmatched | 24.411 | 24.647 | -4.2 | 25.7 | -1.28 | 0.200 |
|  | Matched | 24.396 | 24.571 | -3.1 |  | -0.86 | 0.391 |
| **Birth Order** | Unmatched | 1.8303 | 2.3141 | -37 | 97 | -10.84 | 0.000 |
|  | Matched | 1.8306 | 1.8452 | -1.1 |  | -0.37 | 0.712 |
| **Religion** |  |  |  |  |  |  |  |
| Muslims | Unmatched | 0.90673 | 0.92522 | -6.7 | 98.8 | -2.11 | 0.035 |
|  | Matched | 0.90724 | 0.90701 | 0.1 |  | 0.02 | 0.983 |
| **Maternal Education** |  |  |  |  |  |  |  |
| Primary | Unmatched | 0.18163 | 0.31672 | -31.6 | 93.3 | -9.52 | 0.000 |
|  | Matched | 0.18201 | 0.19101 | -2.1 |  | -0.62 | 0.538 |
| Secondary or higher | Unmatched | 0.76017 | 0.51512 | 52.7 | 96.6 | 15.96 | 0.000 |
|  | Matched | 0.75966 | 0.75137 | 1.8 |  | 0.51 | 0.607 |
| **Husband Education** |  |  |  |  |  |  |  |
| Primary | Unmatched | 0.22596 | 0.3371 | -24.9 | 94 | -7.58 | 0.000 |
|  | Matched | 0.22628 | 0.23292 | -1.5 |  | -0.42 | 0.674 |
| Secondary or Higher | Unmatched | 0.64281 | 0.39063 | 52.1 | 95.2 | 16.16 | 0.000 |
|  | Matched | 0.6423 | 0.63026 | 2.5 |  | 0.67 | 0.504 |
| **Literacy** |  |  |  |  |  |  |  |
| Able to read whole sentence | Unmatched | 0.82398 | 0.60971 | 49 | 96.4 | 14.61 | 0.000 |
|  | Matched | 0.82361 | 0.8159 | 1.8 |  | 0.53 | 0.593 |
| **Wanted Pregnancy** |  |  |  |  |  |  |  |
| Wanted Pregnancy then or later | Unmatched | 0.9467 | 0.875 | 25.4 | 98.8 | 7.41 | 0.000 |
|  | Matched | 0.94659 | 0.94572 | 0.3 |  | 0.10 | 0.918 |
| **Media Exposure** |  |  |  |  |  |  |  |
| Exposed to one media outlet at least once a week | Unmatched | 0.56802 | 0.39182 | 35.8 | 95.7 | 11.17 | 0.000 |
|  | Matched | 0.56922 | 0.56166 | 1.5 |  | 0.41 | 0.684 |
| Exposed to two or three media outlets at least once a week | Unmatched | 0.14165 | 0.03523 | 38.1 | 94.4 | 13.28 | 0.000 |
|  | Matched | 0.14055 | 0.14649 | -2.1 |  | -0.45 | 0.651 |
| **Autonomy** |  |  |  |  |  |  |  |
| Has say in one autonomy indicator | Unmatched | 0.15989 | 0.16783 | -2.1 | 87.4 | -0.66 | 0.506 |
|  | Matched | 0.16022 | 0.16123 | -0.3 |  | -0.07 | 0.942 |
| Has say in two autonomy indicators | Unmatched | 0.15147 | 0.13892 | 3.6 | 2.6 | 1.12 | 0.265 |
|  | Matched | 0.15039 | 0.16261 | -3.5 |  | -0.90 | 0.370 |
| Has say in three autonomy indicators | Unmatched | 0.45302 | 0.40346 | 10 | 81.4 | 3.13 | 0.002 |
|  | Matched | 0.45327 | 0.44406 | 1.9 |  | 0.49 | 0.622 |
| **Location** |  |  |  |  |  |  |  |
| Urban | Unmatched | 0.46073 | 0.25523 | 43.9 | 98.4 | 13.98 | 0.000 |
|  | Matched | 0.45959 | 0.46282 | -0.7 |  | 0.10 | 0.918 |
| **Wealth** |  |  |  |  |  |  |  |
| Intermediate | Unmatched | 0.15428 | 0.20837 | -14.1 | 93.8 | -4.29 | 0.000 |
|  | Matched | 0.1546 | 0.15126 | 0.9 |  | 0.25 | 0.804 |
| Rich and Richest | Unmatched | 0.61571 | 0.31373 | 63.5 | 97.5 | 19.92 | 0.000 |
|  | Matched | 0.6149 | 0.60748 | 1.6 |  | 0.41 | 0.685 |
| **Lost Pregnancy** |  |  |  |  |  |  |  |
| Yes | Unmatched | 0.14727 | 0.11765 | 8.7 | 31.7 | 2.77 | 0.006 |
|  | Matched | 0.14547 | 0.1657 | -6 |  | -1.49 | 0.137 |
| **Skilled ANC Provider** |  |  |  |  |  |  |  |
| Yes | Unmatched | 0.82468 | 0.57394 | 56.8 | 97 | 16.92 | 0.000 |
|  | Matched | 0.82431 | 0.81691 | 1.7 |  | 0.51 | 0.607 |
