# Supplementary material for: Do four or more antenatal care visits increase skilled birth attendant use and institutional delivery in Bangladesh? A propensity-score matched analysis
Source: BMC Public Health. 2019 May 16;19:583. doi: 10.1186/s12889-019-6945-4 (PMC6521440; doi:10.1186/s12889-019-6945-4)
Supplement: Supplementary file 2 — Table S2. Covariate Balance check for Institutional Delivery (DOCX 23 kb) [file 12889_2019_6945_MOESM2_ESM.docx]

**Table S2: Covariate Balance Check for Institutional Delivery use in Bangladesh**

| Variable |  | Mean | | % Bias | % Reduction \|Bias\| | T -Test | |
| --- | --- | --- | --- | --- | --- | --- | --- |
|  |  | Treated | Control |  |  | t | p > \|t\| |
| **Age** | Unmatched | 24.411 | 24.647 | -4.2 | 27.2 | -1.28 | 0.200 |
|  | Matched | 24.4 | 24.572 | -3.1 |  | -0.84 | 0.401 |
| **Birth Order** | Unmatched | 1.8303 | 2.3141 | -37 | 97.2 | -10.84 | 0.000 |
|  | Matched | 1.8318 | 1.8452 | -1 |  | -0.34 | 0.734 |
| **Religion** |  |  |  |  |  |  |  |
| Muslims | Unmatched | 0.90673 | 0.92522 | -6.7 | 95.5 | -2.11 | 0.035 |
|  | Matched | 0.90781 | 0.90698 | 0.3 |  | 0.08 | 0.939 |
| **Maternal Education** |  |  |  |  |  |  |  |
| Primary | Unmatched | 0.18163 | 0.31672 | -31.6 | 93.5 | -9.52 | 0.000 |
|  | Matched | 0.18227 | 0.19107 | -2.1 |  | -0.60 | 0.547 |
| Secondary or higher | Unmatched | 0.76017 | 0.51512 | 52.7 | 96.7 | 15.96 | 0.000 |
|  | Matched | 0.75932 | 0.7513 | 1.7 |  | 0.50 | 0.619 |
| **Husband Education** |  |  |  |  |  |  |  |
| Primary | Unmatched | 0.22596 | 0.3371 | -24.9 | 94.3 | -7.58 | 0.000 |
|  | Matched | 0.2266 | 0.23293 | -1.4 |  | -0.40 | 0.688 |
| Secondary or Higher | Unmatched | 0.64281 | 0.39063 | 52.1 | 95.4 | 16.16 | 0.000 |
|  | Matched | 0.6418 | 0.63028 | 2.4 |  | 0.64 | 0.523 |
| **Maternal Literacy** |  |  |  |  |  |  |  |
| Able to read whole sentence | Unmatched | 0.82398 | 0.60971 | 49 | 96.5 | 14.61 | 0.000 |
|  | Matched | 0.82336 | 0.81586 | 1.7 |  | 0.52 | 0.603 |
| **Wanted Pregnancy** |  |  |  |  |  |  |  |
| Wanted Pregnancy then or later | Unmatched | 0.9467 | 0.875 | 25.4 | 98.8 | 7.41 | 0.000 |
|  | Matched | 0.94652 | 0.94567 | 0.3 |  | 0.10 | 0.921 |
| **Media Exposure** |  |  |  |  |  |  |  |
| Exposed to one media outlet at least once a week | Unmatched | 0.56802 | 0.39182 | 35.8 | 95.6 | 11.17 | 0.000 |
|  | Matched | 0.56932 | 0.56162 | 1.6 |  | 0.41 | 0.679 |
| Exposed to two or three media outlets at least once a week | Unmatched | 0.14165 | 0.03523 | 38.1 | 94.5 | 13.28 | 0.000 |
|  | Matched | 0.14075 | 0.14662 | -2.1 |  | -0.45 | 0.655 |
| **Autonomy** |  |  |  |  |  |  |  |
| Has say in one autonomy indicator | Unmatched | 0.15989 | 0.16783 | -2.1 | 90.1 | -0.66 | 0.506 |
|  | Matched | 0.16045 | 0.16124 | -0.2 |  | -0.06 | 0.955 |
| Has say in two autonomy indicators | Unmatched | 0.15147 | 0.13892 | 3.6 | 4.3 | 1.12 | 0.265 |
|  | Matched | 0.1506 | 0.16261 | -3.4 |  | -0.88 | 0.378 |
| Has say in three autonomy indicators | Unmatched | 0.45302 | 0.40346 | 10 | 83.2 | 3.13 | 0.002 |
|  | Matched | 0.4525 | 0.44419 | 1.7 |  | 0.45 | 0.656 |
| **Location** |  |  |  |  |  |  |  |
| Urban | Unmatched | 0.46073 | 0.25523 | 43.9 | 98.4 | 13.98 | 0.000 |
|  | Matched | 0.45954 | 0.46278 | -0.7 |  | -0.17 | 0.862 |
| **Wealth** |  |  |  |  |  |  |  |
| Intermediate | Unmatched | 0.15428 | 0.20837 | -14.1 | 93.4 | -4.29 | 0.000 |
|  | Matched | 0.15482 | 0.15123 | 0.9 |  | 0.27 | 0.791 |
| Rich and Richest | Unmatched | 0.61571 | 0.31373 | 63.5 | 97.5 | 19.92 | 0.000 |
|  | Matched | 0.61506 | 0.60756 | 1.6 |  | 0.41 | 0.682 |
| **Lost Pregnancy** |  |  |  |  |  |  |  |
| Yes | Unmatched | 0.14727 | 0.11765 | 8.7 | 32.2 | 2.77 | 0.006 |
|  | Matched | 0.14567 | 0.16575 | -5.9 |  | -1.48 | 0.140 |
| **Skilled ANC Provider** |  |  |  |  |  |  |  |
| Yes | Unmatched | 0.82468 | 0.57394 | 56.8 | 96.8 | 16.92 | 0.000 |
|  | Matched | 0.82477 | 0.81683 | 1.8 |  | 0.55 | 0.581 |
